# Thank You to Our 2021 Peer Reviewers

**DOI:** 10.1029/2022GH000639

**Published:** 2022-05-02

**Authors:** Gabriel Filippelli, Rita Colwell, Susan Anenberg, John Balbus, Daniela Ceccarelli, Karen Hudson‐Edwards, Antarpreet Jutla, Chiyuan Miao, Paul Sandifer, Avner Vengosh

**Affiliations:** ^1^ Department of Earth Sciences and Center of Urban Health Indiana University‐Purdue University Indianapolis Indianapolis IN USA; ^2^ Institute for Advanced Computer Studies University of Maryland College Park MD USA; ^3^ Milken Institute School of Public Health George Washington University Washington DC USA; ^4^ National Institute of Environmental Health Sciences Bethesda MD USA; ^5^ Research Executive Agency of the European Commission Brussels Belgium; ^6^ Camborne School of Mines and Environment and Sustainability Institute University of Exeter Exeter UK; ^7^ Department of Environmental Engineering Sciences University of Florida Gainesville FL USA; ^8^ Faculty of Geographical Science Beijing Normal University Beijing China; ^9^ Center for Coastal Environmental and Human Health College of Charleston School of Sciences & Mathematic Charleston SC USA; ^10^ Nicholas School of the Environment Division of Earth and Ocean Sciences Duke University Durham NC USA

## Abstract

- The editors thank the 2021 peer reviewers
- In 2021, GeoHealth benefited from 365 reviews provided by 241 of our peers
- A number of individuals submitted multiple reviews for GeoHealth in 2021

Peer review is at the heart of the scientific endeavor, ensuring that high‐quality discoveries are communicated in effective and impactful ways. As a voluntary and mostly anonymous effort, peer review is often poorly recognized. But it is so valuable to journal Editors, and we are often so impressed by the incredibly detailed, constructive, and informative reviews that we get back from reviewers. In 2021, GeoHealth benefited from 365 reviews provided by 241 of our peers for papers submitted to the journal. Thank you all for being such an important part of the scientific process, advancing the communication of discoveries at the intersections of the environmental and health sciences to improve society.

Thank you to the 241 reviewers who submitted 365 reviews in the journal last year. Individuals in italics provided two or more reviews for GeoHealth during the year.

Abegunde, Linda

Adeyemi, Olusola

Akanda, Ali Shafqat

Alaniz, Alberto

Aryal, Yog

*Asare, Ernest*

*Aung, Poe Poe*

*Baker, Rachel*

Barbera, Marcella

Bhanja, Soumendra

Bhattacharya, Prosun

*Borthwick, Alistair G. L.*

Bruno, Walmer

Bu, Qingwei

Buse, Chris

Caceres Lillo, Dante

Casey, Joan

Censi, Paolo

Chamberlain, Jim

Checkley, Will

Chen, Bin

Chen, Hao

*Chen, Kai*

*Chen, Kaiyu*

*Chen, Yixiang*

*Choi, Joon Yul*

Christian, Aaron

*Cindoruk, Sıddık*

*Coccia, Mario*

Coker, Karen Awura Adjoa

Collier, Tracy

Colwell, Rita

*Conibear, Luke*

Corti, Giuseppe

Cowie, Hilary

Coyte, Rachel

*Crippa, Paola*

Crooks, James

DeFlorio‐Barker, Stephanie

Dev, Vas

Deziel, Nicole

Dodge, Somayeh

*Dorevitch, Samuel*

Doumbia, Thierno

*Downs, Kristen*

*Eccles, Kristin*

Fairley, Jerry

Feng, Rui

Finley‐Brook, Mary

*Fisher, Jared*

Ford, Bonne

Fortner, Sarah

Frolov, Konstantin

Fuhrmann, Jan

Galway, Lindsay

*Gao, Yang*

Gaye, Amadou

*Ghude, Sachin*

Godebo, Tewodros

Gohlke, Julia

*Gorris, Morgan*

*Grady, Sue*

Guimarães, Ricardo

Gupta, Lovleen

*Habre, Rima*

*Hanna, Liz*

Hansen, Birgitte

*Hardi, Tamas*

Havenith, George

Hayes, Katie

Heck, Julia

*Henry, Kevin*

Herrero, Hannah

Hilborn, Elizabeth

*Ho, Steven Sai Hang*

*Hofer, Barbara*

*Hoffman, Kate*

*Hoffman‐Hall, Amanda*

*Horton, Daniel*

*Horwell, Claire*

Hoshiko, Sumi

*Huang, Conghong*

*Huang, Jianping*

Huang, Ling

Huang, Wei

Huang, Xiaochi

*Huang, Yaoxian*

Hursthouse, Andrew

Ijumulana, Julian

Izhitskiy, Alexander

Jalalzadeh Fard, Babak

Jeevarajan, Antony

Jesser, Kelsey

Jiang, Penghui

*Jiang, Xiangyu*

Jin, Xiaomeng

*Johnson, Daniel*

Joseph, Naveen

Jutla, Antarpreet

Kang, Mo‐Yeol

*Kerekes, John*

Kioumourtzoglou, Marianthi‐Anna

*Kodros, John*

Kolok, Alan

*Koplitz, Shannon*

Koulov, Boian

Krauchanka, Julia

*Kuang, Wenhui*

*Kumar, Manish*

*Lacey, Forrest*

*Laidlaw, Mark*

*Larbi, Reuben*

*Lay, Claire*

Lee, Dong‐Wook

*Lemos, Leila*

*Li, Jing*

*Li, Ke*

Li, Liping

*Li, Rui*

Li, Yao

*Lipp, Erin*

*Liu, Jianyu*

*Liu, Yisi*

Loboda, Tatiana

Lombardi, Christina

*Luvall, Jeffrey*

Marengo, Jose A

*Matsushita, Naohiko*

Matz, Carlyn

McDuffie, Erin

McMillan, Benjamin

Méndez‐Lázaro, Pablo

*Messier, Kyle*

*Metz, Carlyn*

Miller, Aubrey

*Montgomery, James A.*

Moreau, John

Moreno Madrinan, Max

Morrissey, Margaret

Mueller, Will

*Munteanu, Gabriela*

*Mutheneni, Srinivasa Rao*

*Nath, Debashis*

*Nath, Reshmita*

Ng, Eric K.W.

*Nobel, Anne*

Noi, Evgeny

*Notari, Alessio*

*Obeng‐Gyasi, Emmanuel*

*O'Dell, Katelyn*

Oguntunde, Phillip

Olusola, Adeyemi

*O'Neill, Susan*

Orimoloye, Israel

Paltseva, Anna

Pan, William

Pant, Pallavi

*Peng, Shushi*

Piketh, Stuart

Pizzitutti, Francesco

*Pleim, Jonathan*

*Propper, Catherine*

Pu, Zhaoxia

Qin, Kun

Qin, Yue

Quist, Arbor

Ransom, Katherine

*Rappold, Ana*

Reid, Colleen

Ren, Jingli

Resnik, David

Restuccia, Francesco

*Reynolds, Heather*

*Ritz, Beate*

Rodriguez‐Villamizar, Laura

*Rogan, Eleanor*

Rohli, Robert

*Rublee, Caitlin*

Ryan, Sadie J

Sacks, Jason

*Saha, Jay*

Saha, Shubhayu

Salamova, Amina

*Sánchez España, Javier*

*Sarofim, Marcus*

Schnell, Jordan

Schullehner, J.

Schwartz, Grace

Scibior, Agnieszka

*Scott, Geoffrey*

Sillé, Fenna

Simeonov, Vasil

Smedley, Pauline

*Smith, Kate*

*Solo‐Gabriel, Helena*

*Song, Yiquan*

*Sorensen, Cecilia*

*Spangler, Keith*

*Spear, Robert*

Spracklen, Dominick

Stowell, Jennifer

*Sun, Zengguo*

Surendran, Anish

Talamantes, Jorge

Tantrakarnapa, Kraichat

Tomašek, Ines

TS, Anish

Turner, Alexander

*Unnikrishnan, Avinash*

*Upadhyay, Abhishek*

*Varotsos, Konstantinos*

*Von Borries, Rosa*

Wade, Anna

*Wang, Jin‐Feng*

Wang, Pengfei

Wang, Suwei

Wang, Xuming

Weaver, Elizabeth

Weber, Rodney

*Weinberger, Kate*

Wells, Ellen

*Wimberly, Michael*

Wingo, Jonathan

*Wood, Leah*

Wu, Bobo

*Xiao, Xiao*

Xin, Jinyuan

You, Yongfa

Yu, Dayang

Yu, Hanchen

Yu, Le

Yu, Manzhu

*Yue, Yao*

Yusuf, Marthins

*Zaitchik, Benjamin*

*Zhao, Chuanfeng*

Zhou, Mengjie

Zhu, Likai

Zohny, Sarah

*Zou, Yufei*

Zuddas, Pierpaolo

## Conflict of Interest

The authors declare no conflicts of interest relevant to this study.

